# Bayesian Weighting of Statistical Potentials in NMR Structure Calculation

**DOI:** 10.1371/journal.pone.0100197

**Published:** 2014-06-23

**Authors:** Martin Mechelke, Michael Habeck

**Affiliations:** 1 Institute for Mathematical Stochastics, Georg August University Göttingen, Göttingen, Germany; 2 Department of Protein Evolution, Max Planck Institute for Developmental Biology, Tübingen, Germany; Duke University Medical Center, Duke University, United States of America

## Abstract

The use of statistical potentials in NMR structure calculation improves the accuracy of the final structure but also raises issues of double counting and possible bias. Because statistical potentials are averaged over a large set of structures, they may not reflect the preferences of a particular structure or data set. We propose a Bayesian method to incorporate a knowledge-based backbone dihedral angle potential into an NMR structure calculation. To avoid bias exerted through the backbone potential, we adjust its weight by inferring it from the experimental data. We demonstrate that an optimally weighted potential leads to an improvement in the accuracy and quality of the final structure, especially with sparse and noisy data. Our findings suggest that no universally optimal weight exists, and that the weight should be determined based on the experimental data. Other knowledge-based potentials can be incorporated using the same approach.

## Introduction

Structural data measured by NMR spectroscopy are never complete. Even the most carefully collected data will by themselves not allow us to determine the three-dimensional structure of a biomolecule with atomic resolution. Rather, we need to interpret the data in the light of prior knowledge that is typically encoded in a potential function or force field [1].

Potential functions quantify the forces and interactions within a biomolecule and with its environment. Two fundamentally different approaches of designing potential functions are commonly used [2]. Physics-based force fields [3] aim to approximate the underlying physical laws. Statistical or knowledge-based potentials [4] are learned from a structure database and describe the effective forces resulting from all interactions including those with the solvent. Physical and statistical potentials are complementary in the sense that some interactions cannot be broken down easily into fundamental, physical contributions but are captured more effectively by potentials derived from known structures.

In NMR structure calculation, potential functions are used to guide the calculation towards structures of high quality and accuracy [1]. This guidance is needed because NMR measurements by themselves do not allow us to determine the three-dimensional structure of an entire macromolecule. To “let the data speak for themselves” and also for reasons of computational efficiency, one tends to use minimalist force fields that ignore complex effects such as electrostatic screening or solvent interactions. If additional potentials such as dihedral angle [5], [6] or hydrogen bonding potentials [7] are used, their force constants are set *ad hoc* and held fixed during the structure calculation.

However, it might be necessary to adjust the force constants for each data set. Because knowledge-based potentials represent averages over large sets of structures, they are not universally transferable and may not represent the preferences of a particular structure. Think of a backbone dihedral angle potential, a “Ramachandran potential”, as an example. The minimal energy configuration of the Ramachandran potential is completely alpha-helical. Therefore it seems more appropriate to choose a higher weight for helical proteins than for all-beta proteins.

Here we introduce an objective, data-driven approach to find the optimal force constant for a given protein and data set. Our method is based on statistical mechanics and Bayesian inference and allows us to incorporate knowledge-based potential functions without biasing the structure calculation.

## Results

### Statistical potential for backbone dihedral angles

**Figure 1:**
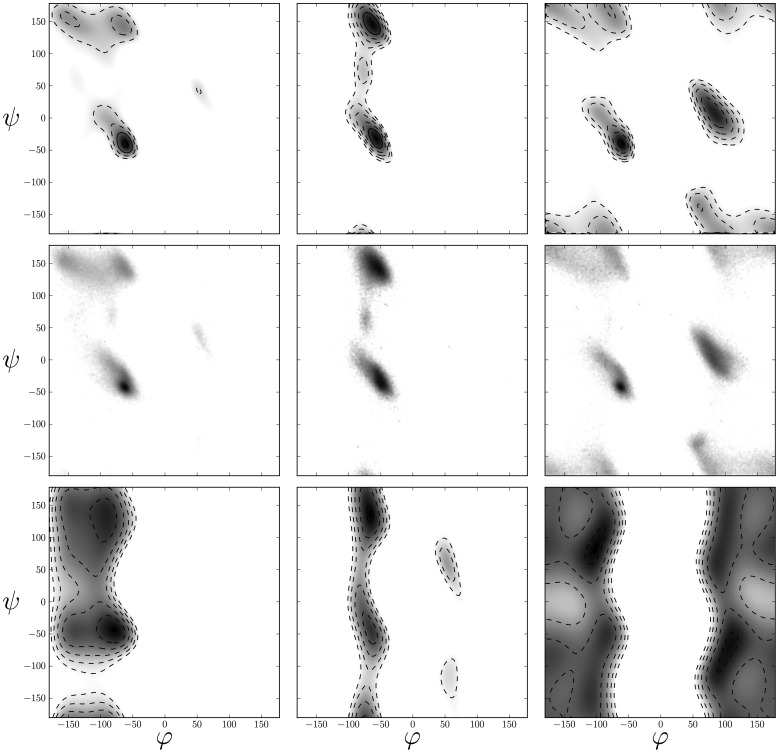
Backbone dihedral angle distributions of amino acids estimated from high-resolution crystal structures. Density maps of 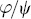 distributions for Alanine (left column), Proline (middle column) and Glycine (right column) as approximated by the maximum entropy distribution (top row). The middle row shows the empirical 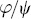 distribution computed over a non-redundant structure database. The bottom row shows the 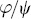 distribution obtained by sampling structures from the nonbonded force field.

Protein backbone dihedral angles 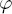 and 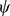 show a typical correlation pattern, an observation made by Ramachandran *et al.* [8] assuming only hard-sphere steric repulsion between atoms. Standard nonbonded energies used in NMR structure determination [9] do not fully capture all aspects of 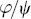 distributions observed in high-resolution crystal structures [10].The dihedral angle distributions obtained from nonbonded interactions do not reproduce the empirical distribution (see the bottom row of Figure 1 and Figures S2–S4 in File S1). Depending on the quality of the data, NMR structures can show dihedral angles outside the allowed regions of the Ramachandran plot [11], [12]. For this reason it is common practice [13] to assess the Ramachandran statistics of NMR structures by programs such as Procheck [14], MolProbity [15] and WhatCheck [16]. To obtain more regular NMR structures, various dihedral angle potentials derived from database statistics have been developed [5], [6]. The functional forms of these potentials range from two-dimensional histograms [17] to continuous representations based on linear interpolation, cubic splines and statistical density estimation [18]–[20]. Some of these models ignore that 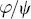 distributions are smooth and periodic, which can result in artifact in the refinement [21].

We use nonparametric density estimation to derive a backbone potential and expand the joint 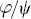 distribution into a Fourier series [22]. This representation is inherently smooth and periodic and has the advantage that it can easily represent multimodal distributions. Each distribution is a linear combination of 80 two-dimensional cosine and sine functions resulting from the combination of five frequencies in the 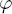 and 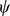 dimensions. The estimated distributions capture features such as the alpha-helical peak and regions corresponding to parallel and anti-parallel beta sheets. Also rare secondary structures such as left-handed helices are represented accurately. Figure 1 shows the estimated dihedral distribution of three representative amino acids and the corresponding empirical histograms (see Figure S2 in File S1 for the full set of dihedral angle distributions).

### Data-driven weighting of the backbone potential

We use the probabilistic Inferential structure determination approach (ISD) [23], [24] to determine protein structures from experimental data. In a standard ISD calculation, one explores the posterior probability 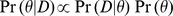 of all conformational degrees of freedom 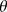 given the experimental data 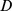. The posterior distribution itself is proportional to the likelihood function 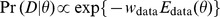 and the prior probability 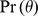. The likelihood is the probability of the data given the structure and involves 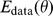, a measure of the goodness-of-fit between the data and a particular structure 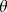; the weight 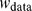 allows us to balance the data against the prior probability. The prior distribution is typically a Boltzmann distribution at inverse temperature 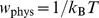, 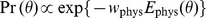 resulting from the force field 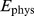. We incorporate the newly derived backbone potential 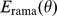 by extending the prior distribution: 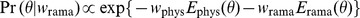 where 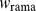 is the weight of the backbone potential.

**Figure 2:**
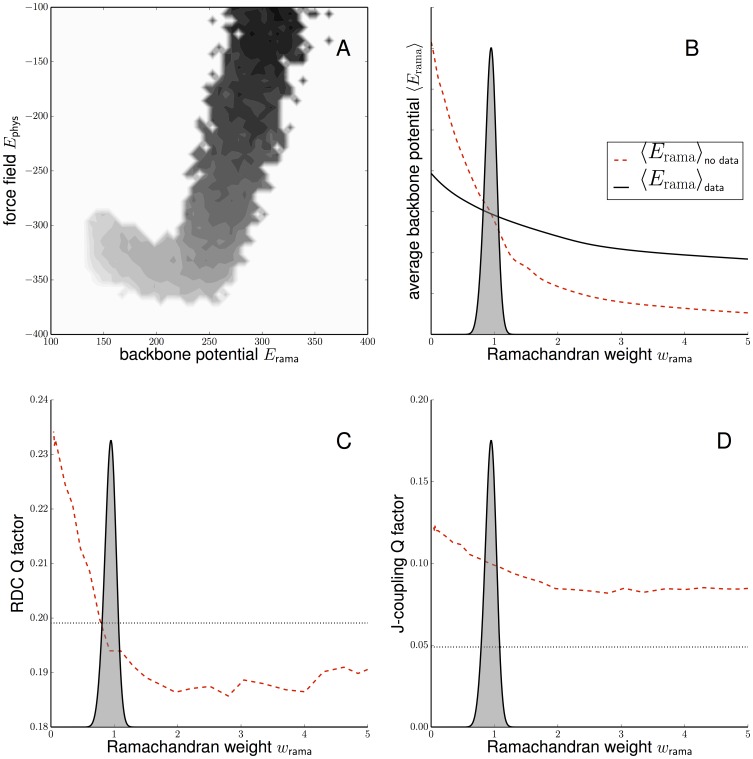
Bayesian weighting of the backbone potential for ubiquitin inferred from high-quality distance data. A: Correlation between backbone potential and nonbonded force field. Shown is the joint distribution of physics- and knowledge-based contributions in the absence of any structural data. (The energies of the crystall structure are 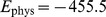 and 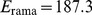.) B: Model evidence 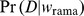 as a function of the Ramachandran weight 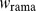. C: Influence of the Ramachandran weight on the average Q-factor (red dashed line) calculated for 11 RDC data sets that were not used in the structure calculation. The Q-factor reflects the agreement between experimental and calculated RDCs. The dotted black line indicates the average Q-factor of the crystal structure (PDB code: 1ubq). D: Influence of the Ramachandran weight on the fit with scalar coupling measurements (red dashed line). Six three-bond scalar coupling data sets are available for ubiquitin and have not been used in the structure calculation. The dotted black line indicates the average Q-factor of the crystal structure (PDB code: 1ubq). The grey distribution indicates the model evidence 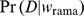.

The weight of the backbone potential 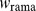 is unknown and has to be chosen somehow. Naively, we would set it to one (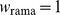). But this is problematic because some aspects of the Ramachandran plot are already captured by the force field. Figures 1 and S3 in File S1 show that structures calculated on the basis of the nonbonded force field 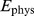 already reproduce the rough outline of the 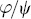 basins. But there are more subtle aspects such as optimal hydrogen bonding geometry [25] that result in pronounced peaks, which are not reproduced by the force field alone. As a consequence, the force field and the backbone potential are not independent of each other but are positively and negatively correlated depending on the energy range (Figure 2A). Setting the 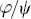 weight to a large value risks that we overemphasize these contributions in the combined potential. In the limiting case, we will force the structure into a helical conformation, whereas with too small 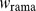 the effect of the backbone potential becomes negligible. Therefore, we need to adjust 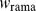 according to the experimental data and the structure.

We have introduced a Bayesian approach to estimate the weight of the experimental data 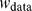 relative to the prior probability [26]. This approach exploits the fact that for every conformation we can calculate how well it agrees with the data and that its goodness-of-fit determines the weight of the data 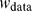. The same is not possible for 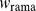, because the statistics reflected by the backbone potential recapitulate an ensemble property, and therefore we need to assess how well the entire *ensemble* agrees with the Ramachandran statistics. Thus it is computationally much more demanding to adjust 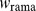 than 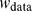.

To estimate 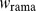, we compare the differences between the expected backbone energy 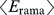 where 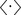 denotes an ensemble average. 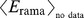 summarizes how the force field and the backbone potential are correlated independent of any data. To obtain this ensemble average, structures are sampled based on the combined energy 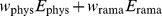. This value is contrasted with the expected backbone energy obtained with data 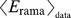. To calculate this ensemble average, structures are sampled based on the full energy 
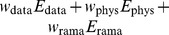
. If 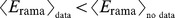, the addition of the experimental data improves the backbone energy, and we can increase 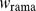 because the data comply with the Ramachandran statistics. If the data contradict the backbone potential, 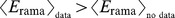, we must lower the weight because the backbone potential biases the ensemble. The optimal weight achieves 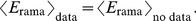
(1)


This rule can be derived rigorously [27] by maximization of the *model evidence*, 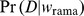, which is the probability of observing the data for a particular value of 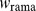 and whose computation involves an ensemble average. It can be shown that the derivative of the model evidence is the difference between the average Ramachandran potential under the posterior and the prior (i.e. with and without data) [27]. At the maximum evidence the derivative vanishes, therefore both energies are the same and the curves cross. If we choose the weight from this region, we bias our ensembles the least.

### Bayesian weighting with high-quality data

We used Bayesian weighting to analyze the high-quality data for ubiquitin (PDB code: 1d3z). We estimated the optimal weight from the NOE-based distances and used the additional scalar and dipolar data for validation. Figure 2B shows the model evidence 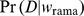, which peaks at the optimal weight satisfying equation (1). Assuming a uniform prior probability for 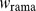, the estimated weight of the backbone potential is 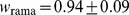. An additional control for evaluating the Bayesian choice of the Ramachandran weight are the residual dipolar couplings (RDCs) and scalar coupling measurements that are available for ubiquitin. For each of the 11 RDC sets, we calculated the average Q-factor obtained for different choices of 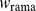. Figure 2C shows that the Bayesian choice of the Ramachandran weight improves the Q-factor to 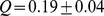. Similarly, we see an improvement in the fit with the scalar coupling measurements (Fig. 2D).

### Bayesian weighting with incomplete data

**Figure 3:**
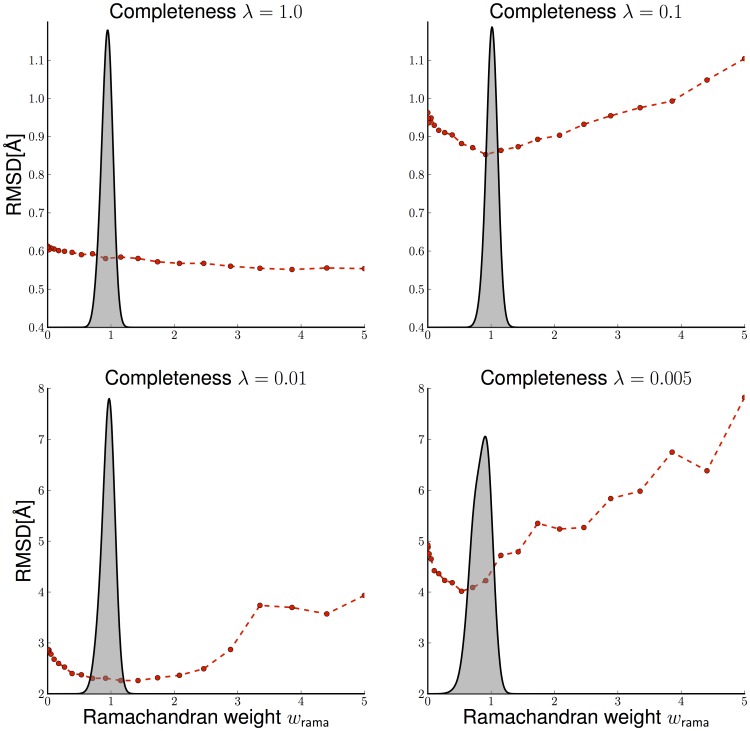
Impact of incomplete ubiquitin data on 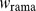. Shown is the model evidence as a function of 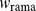 (grey) and the average RMSD (dots). The sparsity increases from the top left panel to the bottom right panel.

Next, we studied how the weight changes for sparse versions of the ubiquitin data. To do so, we introduce a completeness parameter 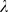, which controls the amount of data [28]. For 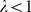, the effective number of observations is reduced. As observed before [27], the model evidence broadens and shifts towards smaller values if we reduce the number of observations (Fig. 3). The estimated weights are: 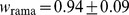 (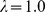), 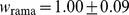 (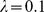), 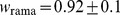 (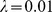), and 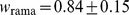 (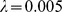). If we reduce the number of data further, the system undergoes a phase transition because the posterior no longer peaks at the native ensemble, and 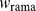 is pushed toward zero. For all values of 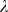, the optimal weight falls in the range that results in a minimal RMSD to the crystal structure. The Bayesian choice of 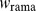 generates the most accurate structures.

### Impact on structure ensembles from sparse and noisy NMR data

So far, we studied how Bayesian weighting of the backbone potential impacts the conformational ensemble under artificially sparsified data. We also looked at challenging real-world structure determination problems, a sparse set of distances for the Fyn-SH3 domain [23], [29] and noisy distance bounds measured with solid-state NMR on the 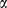-spectrin SH3 domain [30]. We estimated the Ramachandran weight for both data sets and obtained 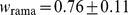 and 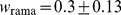 for the sparse and noisy distances, respectively (see also Figure S7 in File S1).

**Figure 4:**
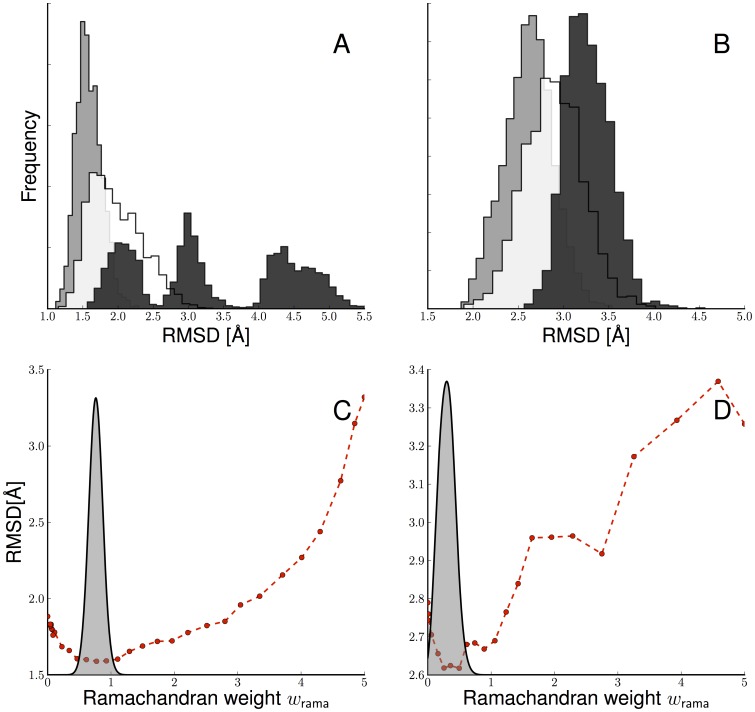
Impact on structure ensembles from sparse and noisy NMR data. Panels A, C show the results for the sparse Fyn-SH3 data set. Panels B, D show the results for the solid-state data. The top row displays the RMSD distributions with 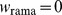 (white), 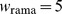 (black) and optimal 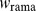 (grey). The grey distribution shown in the bottom panels is the model evidence as a function of the weight 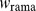.

Incorporation of the backbone potential can significantly improve the accuracy of the ensemble as measured by the RMSD to the crystal structure (Figure 4). But we also observe that an overly strong backbone potential can do more harm than good. For large 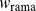 values, the RMSD distribution deteriorates and even shows multiple peaks in case of the sparse data set. With an optimally weighted backbone potential also the accuracy of the mean structure is consistently higher than the accuracy of the individual members of the structure ensemble (see Table S1 in File S1). This indicates that the structure ensembles are better defined when using the backbone potential. Figure 4 shows that the model evidence peaks where the average RMSD to the crystal structure shows a minimum.

**Figure 5:**
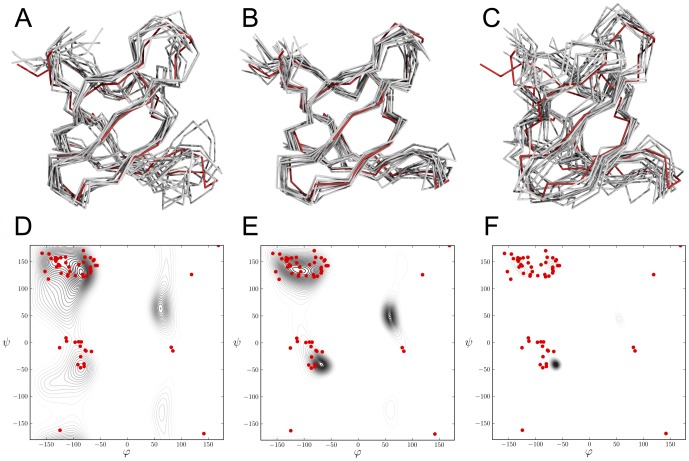
Influence of the weight 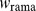 on the structural ensemble of Fyn-SH3 inferred with sparse NMR data. Shown are the conformations and backbone dihedral distributions generated with different 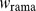. Panels A–C display structure ensembles comprising ten randomly selected conformations (grey) superimposed onto the crystal structure (red). Panels D–F show in black a maximum entropy distribution fitted to the backbone torsion angles of the structures generated with ISD. The backbone dihedral angles of the crystal structure are marked by red dots. Panels A, D show the results for 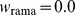, panels B, E: 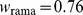 (optimal weight), panels C, F: 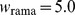 (maximum weight probed during replica-exchange simulations).

Figure 5 shows the structure ensembles obtained with the sparse SH3 data for different choices of 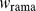. If the weight is zero or too small, the ensemble is still quite heterogeneous, especially in the loops. When incorporating the backbone potential with an optimal weight, the ensemble becomes very regular and accurate: the average structure is surprisingly close to the reference structure (1.05 Å RMSD) given the sparseness of the data set. For too large weight, we introduce conflicts between the preferences of the statistical potential and the data by introducing helical structure in beta strands. The corresponding Ramachandran plots illustrate these findings. For 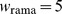, the Ramachandran plot becomes artificially narrow and peaks in the helical region. Another indication that the Bayesian choice of 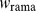 is optimal, is provided by the behavior of additional model parameters. Figure S8 in File S1 shows that the estimated weight of the data 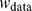 [26] is largely unaffected if we incorporate the knowledge-based contribution. That is, we do not compromise the data by downweighting them upon integration of the backbone potential.

**Figure 6:**
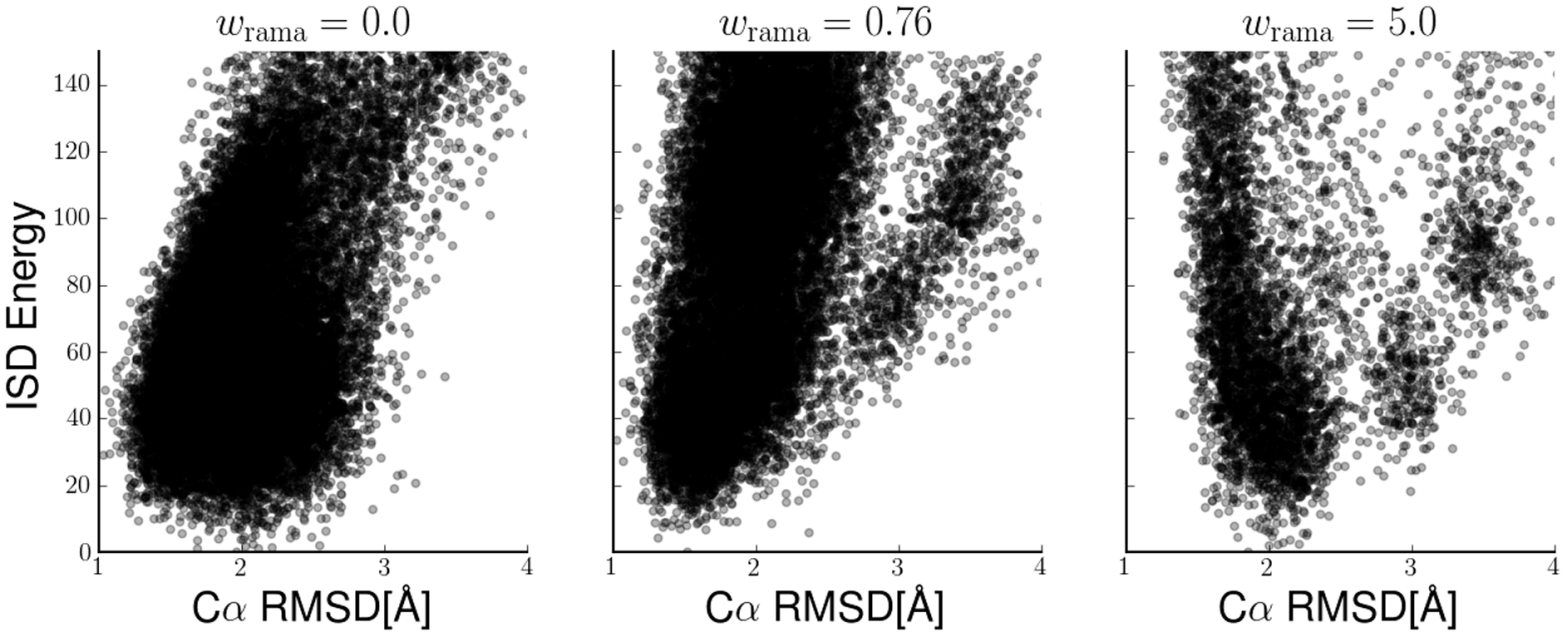
Energy funnels obtained with the sparse Fyn-SH3 data at different Ramachandran weights (left: 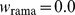, middle: 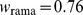, right: 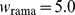). The full ISD energy (negative log-posterior probability) is plotted against the RMSD to the Fyn-SH3 crystal structure.

In Figure 6 we plot the effective potential function (i.e. the negative log-posterior probability) incorporating the force field, the backbone potential and the goodness-of-fit for different values of 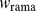 corresponding to the ensembles shown in Figure 5. The results indicate that optimal weighting helps to guide the simulation towards more accurate structures. Without the backbone potential, structures at the bottom of the energy funnel show a broad range of RMSDs between 1.5 and 2.5 Å. For optimal 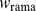, the funnel narrows and selects structures with an RMSD below 1.5 Å. With too large 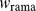 we observe a negative correlation between the RMSD and the negative log-posterior probability.

### Impact on structure quality

**Figure 7:**
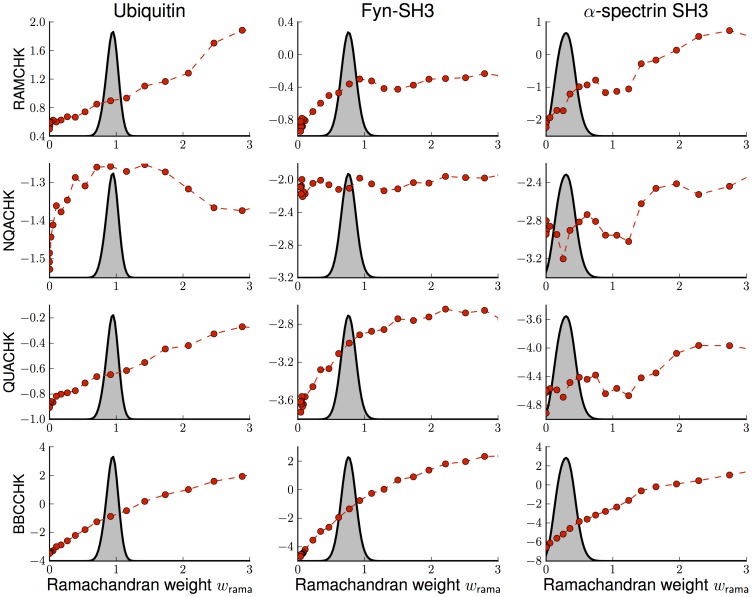
Influence of the Ramachandran weight on various quality criteria. Shown is the impact of 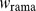 on WhatCheck validation criteria. Each column reports the results for a different data set (left column: ubiquitin, middle column: Fyn-SH3 domain, right column: 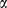-spectrin SH3 domain). Each row shows the evolution of a quality score with increasing 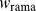 (each dot marks the average over 100 structures that were randomly selected from the ISD ensemble, dashed lines are added to guide the eye). The first row reports the Ramachandran appearance as assessed by RAMCHK. The second and third row show WhatCheck's packing scores. The last row reports the regularity of the backbone (BBCCK). The grey distribution indicates the model evidence 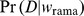 as a function of 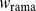.

Figure 7 shows the average values of several validation criteria for structures generated at different weights (a full report of the Procheck and WhatCheck quality criteria can be found in Table S1 in File S1). All reported criteria are z-scores that provide an assessment of how a particular structure compares to the average of all known protein structures in terms of standard deviations. That is, a z-score of 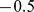, say, means that the quality criterion of this particular structure is half a standard deviation below the average.

As expected, the Ramachandran score (RAMCHK) improves with increasing 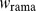 and shows no saturation, which demonstrates that it is not a valid indicator for selecting 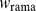. The effect of the backbone potential on the NQACHK score is small; only for ubiquitin it agrees with the Bayesian choice of 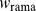. WhatCheck's packing score QUACHK shows a different behavior, it increase steadily with increasing 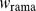. BBCCHK assesses the regularity of the backbone and shows a steady increase with increasing 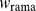 indicating a high correlation with the potential Ramachandran potential. Although the ubiquitin data are of a high quality, the structure ensemble still improves upon incorporation of the backbone potential. As for ubiquitin, we observe an overall improvement in the quality of the ensembles obtained with the sparse and noisy SH3 data. The slight decrease of the average NQACHK score for the optimal weight ensemble in the case alpha-spectrin is within the ensemble spread; again the score is only weakly affected by the Ramachandran weight (see Figure S5 in File S1).

The WHATCHECK validation criteria by themselves are unable to choose a 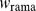 that would lead to a global improvement of the structural quality as well as the accuracy of the ensemble. Moreover, although some of the scores seem to be highly correlated (e.g. RAMCHK and BBCCHK, see Figure S6 in File S1) it is not clear whether it is possible to maximize all scores simultaneously. Rather we have to find a comprise between the different quality criteria, and this is exactly what our weighting scheme achieves.

## Discussion

We outline a new formalism to integrate physics- with knowledge-based potential functions in biomolecular structure calculation. Our approach is founded on Bayesian principles and allows us to incorporate prior knowledge derived from structure databases. The new method is data-driven and adaptively weighs knowledge-based contributions relative to the force field and the experimental data thereby reducing potential bias and artifact. We show that even simple terms such as knowledge-based backbone potentials have a significant effect on the quality of the structure ensemble. The optimally weighted dihedral angle potential improves not only the Ramachandran appearance but also the backbone normality and packing scores. Moreover, it systematically produces more accurate and more precise structure ensembles. The combination of physics- and knowledge-based potential functions is particularly powerful for sparse and noisy NMR data and shifts the ensemble closer to the native structure. Our findings suggest that there is no universal weight that can be transferred to all proteins and data sets. Rather, the method of choice is to estimate the weight in the course of the structure calculation.

For the solid-state SH3 data, we find the smallest weight 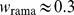; for the ubiquitin data we estimate the largest weight 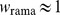. We can rationalize this behavior as follows. For high-quality data (high completeness, low noise level), the optimal weight adopts a large value and drops as noise and sparseness increase [27]. This behavior seems counterintuitive at first sight but is sensible: The forces that pull the ensemble towards the correct structure are weaker with low-quality than with high-quality data. We therefore have to soften the backbone potential to not overwhelm the data.

In the future, we plan to extend our method to weigh multiple statistical energy terms simultaneously in the course of a structure calculation. However, this will require a more efficient algorithm for estimating multi-dimensional densities of states, because the computational complexity of the approach becomes prohibitive. The naive extension of the presented approach would involve a multi-dimensional replica-exchange simulation in which replicas are introduced for every combination of the weights that we want to estimate. Therefore the computational burden grows exponentially in the number of weights such that with our current algorithm it is only possible to estimate up to two or three weights. The final goal is to design an efficient, unbiased but highly informative conformational prior distribution that allows the calculation of high quality ensembles from very sparse data sets.

## Materials and Methods

### Data sets

Backbone dihedral angles were extracted from PDBselect25 [31] and used to estimate angular distributions for all amino acids using the maximum entropy method (see next section). We illustrate the impact of the backbone potential on three NMR data sets. The first data set (PDB code: 1d3z) comprises high-quality data for ubiquitin. All distance data were reduced to 1444 non-redundant restraints, additional data (scalar coupling constants, residual dipolar couplings) were not included in the structure calculation but used for validation. The second data set comprises sparse distance data for the Fyn-SH3 domain [23], [29] (PDB code: 1zbj). The third data set has been measured with solid-state NMR on the 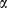-spectrin SH3 domain [30] (PDB code: 1m8m). The solid-state data are very generous distance bounds ranging from 4.5 Å to 7.5 Å out of which 90% are equal or greater than 6 Å, which is the largest distance bound obtained in standard solution NMR.

### Maximum entropy distributions for backbone dihedral angles

Following Pertsemlidis *et al.* [22], we use a maximum entropy distribution with a Fourier basis to describe the distribution of backbone dihedral angles: 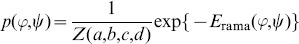
(2)where the Ramachandran potential 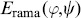 is given by



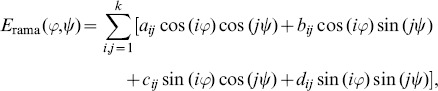
(3)

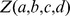
 normalizes the dihedral angle distribution, and 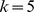 is the order of the Fourier expansion (see Figure S1 in File S1). We fit the expansion coefficients 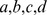 to observed 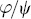 pairs by using the maximum entropy method, which, in this case, is equivalent to maximum likelihood parameter estimation. We approximate the normalization constant 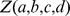 using the two-dimensional trapezoidal rule. To avoid over-fitting of the 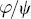 distributions, we introduce a Gaussian prior with unknown precision 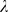 over the expansion coefficients: 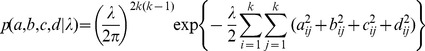
(4)


The precision of the prior 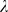 is not known and is estimated simultaneously with the expansion coefficients. We use an iterative scheme in which we cycle through updates of the expansion coefficients and of the precision. For fixed precision, the negative log-posterior probability of the expansion coefficients is a convex function, which we optimize using the Powell minimizer [32]. The update of the precision can be calculated analytically.

### Inferential structure determination

Inferential structure determination (ISD) [23], [24] is a probabilistic framework for biomolecular structure calculation from experimental data. ISD uses Bayes's theorem to obtain a posterior distribution over all unknown parameters including the conformational degrees of freedom 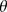 (typically main and side chain torsions) and all additional model parameters 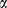 [33]. The posterior distribution is proportional to the product of two terms, the likelihood function and the prior probability distribution. The likelihood function, 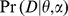, is the conditional probability of the experimental data 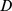 viewed as a function of the parameters 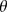 and 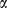. Here we consider NMR distance measurements, which we model using the lognormal distribution [34]. This introduces two model parameters, the distance scale and error, that we estimate simultaneously with the structure. We use the lognormal model for the analysis of the 1d3z and 1zbj data, which provide distance restraints. In case of the solid-state data (PDB code: 1m8m), only lower and upper distance bounds are given. We apply a new probabilistic model (Habeck, in preparation) that estimates a set of experimental distances falling between the lower and upper bounds. For given estimated experimental distances, the lognormal model is used to relate the distance data with the structure. This model has in addition to the set of unknown experimental distances only one model parameter, the distance error.

### Optimization of the potential function

We aim to find an optimal potential function for each dataset by adjusting the influence of the backbone potential 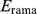. The combined potential function is given by 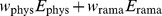 where 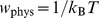 is the reciprocal temperature involving Boltzmann's constant 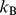 and the absolute temperature 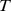. Here 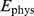 was set to the Lennard-Jones potential adapted from the Rosetta software [35]. The only free parameter is the weight of the backbone potential 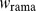. The model evidence 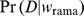 can be interpreted as the probability of the experimental data for a particular 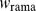. The optimization of 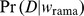 is demanding as the calculation depends an intractable high-dimensional integral: 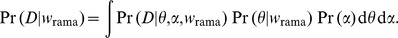
over all model parameters 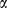 and conformational degrees of freedom 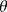. Here 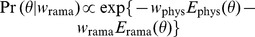 is the combined prior probability of conformation 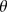 for a given weight 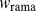. We can reduce the computation to a low-dimensional integral by expressing the above equation using the density of states 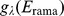


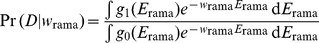



The density of states is given as 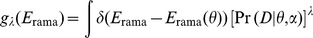





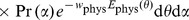
where 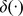 denotes the Dirac delta function. Estimates of the density of states are obtained by applying multiple histogram reweighting [36], [37] as outlined in [27].

### Replica-exchange Monte Carlo

To estimate the density of states we generate conformations using an extended replica-exchange Monte Carlo scheme (REMC). The idea behind REMC is to simulate a system in parallel at different temperatures [38]. In our scheme, two temperature parameters control the force field and the likelihood function independently [39]. We treat 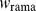 as a third temperature parameter. Across the first 30 replicas 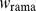 decreases from five to zero while the force field and the data are fully taken into account. In the remaining 50 replicas, the force field and the data are gradually switched off as described by Habeck *et al.* [39]. The convergence of the method depends on the size of the system as well as the quality and quantity of the data. In case of the tested systems, convergence was achieved after 30000 to 100000 replica transitions corresponding to 2–3 days worth of computation on a 80 node cluster depending on the size of the system.

## Supporting Information

File S1 **Supporting file including supporting text, Figures S1–S8, and Table S1.**
(PDF)
