# Supplementary material for: Bayesian Weighting of Statistical Potentials in NMR Structure Calculation
Source: PLoS One. 2014 Jun 23;9(6):e100197. doi: 10.1371/journal.pone.0100197 (PMC4067304; doi:10.1371/journal.pone.0100197)
Supplement: File S1 — Supporting file including supporting text, Figures S1–S8, and Table S1. (PDF) [file pone.0100197.s001.pdf]

Supporting information:

Bayesian weighting of statistical potentials in NMR structure calculation

Martin Mechelke [1,2] and Michael Habeck [1]
[1]Institute for Mathematical Stochastics, Georg August University Göttingen, Goldschmidtstrasse 7, 37077 Göttingen, Germany
[2]Department of Protein Evolution, Max Planck Institute for Developmental Biology, Spemannstrasse 35, 72076 Tübingen, Germany

1 Backbone dihedral angle distributions

Empirical energy functions for backbone dihedral angles, "Ramachandran potentials", have been used in biomolecular structure calculation since almost two decades. Several statistical models have been proposed ranging from two-dimensional histograms (Gong et al., 2007) to continuous representations based on linear interpolation, cubic splines and Gaussian mixtures. These models ignore two fundamental aspects of statistical distributions over angular variables, namely their periodicity and their smoothness, which can result in artifacts in the refinement (Kuszewski and Clore, 2000). An alternative is provided by statistical density estimation (Mardia et al., 2007; Boomsma et al., 2008; Ting et al., 2010).

We follow a non-parametric approach based on the principle of Maximum Entropy (Jaynes, 1957). As proposed by Pertsemlidis et al. (2005), we use a Fourier basis to represent the joint distribution of φ and ψ backbone dihedral angles. This representation is inherently smooth and periodic and has the advantage that it can easily cope with multi-modality (as opposed to the unimodal von Mises or Kent distribution which need to be combined into mixtures (Mardia et al., 2007; Boomsma et al., 2008; Ting et al., 2010) in order to represent the multi-modal Ramachandran plot). The functional form of the distribution of backbone dihedral angles is given by:

$$p(\varphi, \psi) = \frac{1}{Z(a, b, c, d)} \exp\{-E(\varphi, \psi)\}, \quad Z(a, b, c, d) = \int \exp\{-E(\varphi, \psi)\}\, d\varphi d\psi \tag{1}$$

where the Ramachandran potential $E(\varphi, \psi)$ is given by:

$$E(\varphi, \psi) = \sum_{i=0}^{k} \sum_{j=0}^{k} \{a_{ij} \cos(i\varphi) \cos(j\psi) + b_{ij} \cos(i\varphi) \sin(j\psi) + c_{ij} \sin(i\varphi) \cos(j\psi) + d_{ij} \sin(i\varphi) \sin(j\psi)\} \tag{2}$$

and $Z(a, b, c, d)$ normalizes the dihedral angle distribution; k is the order of the Fourier expansion. We fit the expansion coefficients a, b, c, d to pairs of φ and ψ angles, extracted from a set of 850 proteins (Dunbrack, 2002) by Maximum Likelihood. During the optimization procedure, we need to evaluate the normalization constant $Z(a, b, c, d)$. Because there is no closed form expression for this integral we evaluate it numerically using the two-dimensional trapezoidal rule. To circumvent overfitting of data and to encourage sparse parameter sets, we introduce a Gaussian prior probability with unknown precision λ over the expansion coefficients a, b, c, d:

$$p(a, b, c, d|\lambda) = \left(\frac{\lambda}{2\pi}\right)^{2k(k-1)} \exp\left\{-\frac{\lambda}{2} \sum_{i=0}^{k} \sum_{j=0}^{k} (a_{ij}^2 + b_{ij}^2 + c_{ij}^2 + d_{ij}^2)\right\} \tag{3}$$

The precision of the prior λ is not known and is estimated simultaneously with the expansion coefficients. We use an iterative scheme in which we cycle through conditional updates of the expansion

coefficients and of the precision. For fixed precision, the log-posterior probability of the expansion coefficients is a convex function, which we optimize using the Powell minimizer (Press et al., 1989). The update of the precision for given a, b, c, d can be calculated analytically.

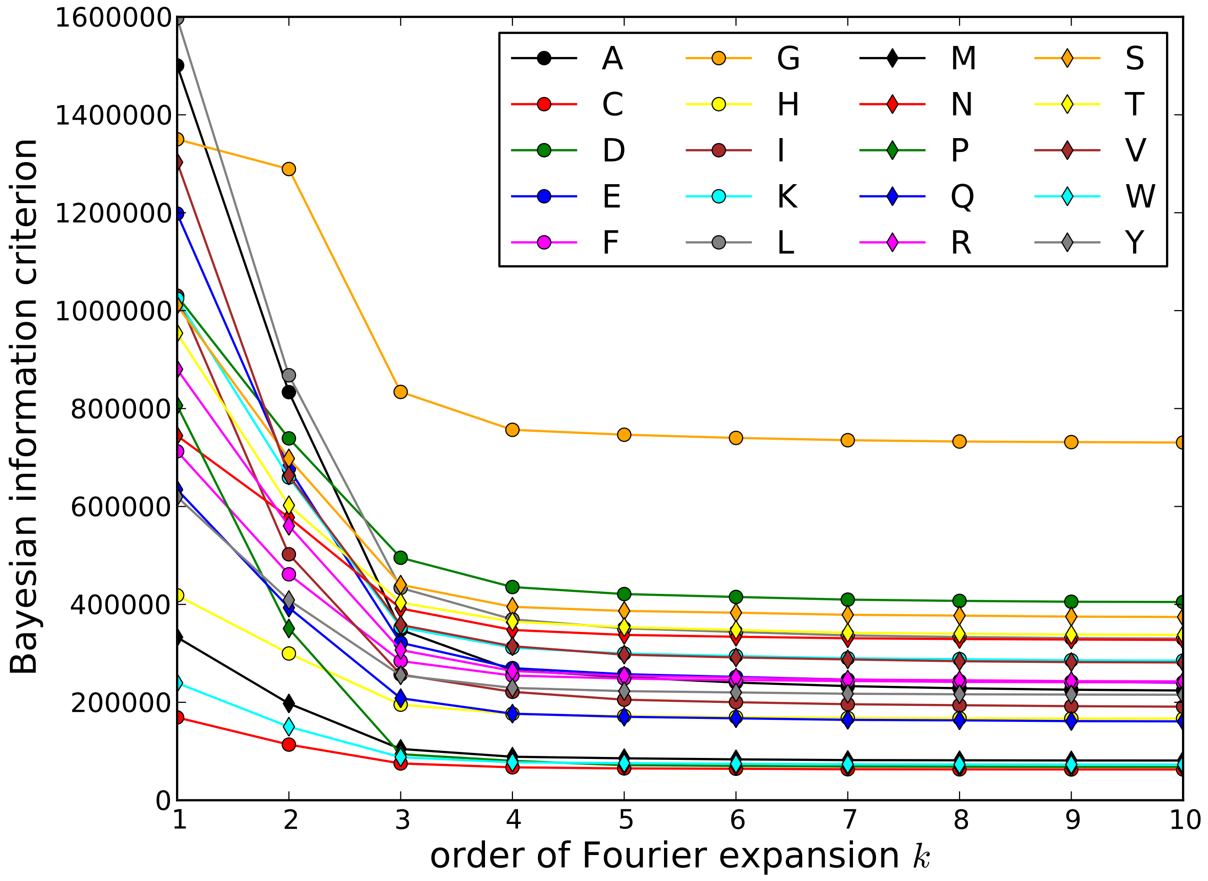

Figure S1: **BIC analysis to determine the optimal order of the Fourier expansion.** The BIC curves of the backbone dihedral angle distributions of all twenty amino acids (indicated as one letter codes in the legend) is shown with increasing Fourier order.

To avoid over-fitting of dihedral angle distributions, we truncate the Fourier series at small orders k. To determine the optimal order of the Fourier expansion we apply the Bayesian information criterion (BIC) (Schwarz, 1978). BIC is a general quantity to select the optimal number of parameters m given data of size n and is defined as

$$\mathrm{BIC}(m) = -2 \log \mathcal{L}_{max} + m \log n$$

where $\mathcal{L}_{max}$ is the maximum of the likelihood of the data set for m parameters (here $m = 4k(k-1)$). The model with the lowest BIC is considered the most probable one and describes the data best. We fitted distributions with Fourier order ranging from 1 to 10 and computed the resulting BIC (Figure S1). We observe that for all amino acids, BIC plateaus at an order of four to five, in accordance with Pertsemlidis et al. (2005). Based on the BIC analysis we fix the maximum order of the Fourier series to five, which amounts to 80 expansion coefficients and results in a good fit that can be evaluated very efficiently and adds negligible computational cost.

Figure S2 shows the estimated dihedral angle distributions of all amino acids. Each distribution is a linear combination of 80 two-dimensional planar waves with different combinations of five frequencies. The resulting estimates capture features such as the α-helix peak at $\varphi = -60°, \psi = -40°$ and

regions corresponding to parallel and anti-parallel β-sheets. Also rare secondary structure elements including left-handed helices are well represented by the Maximum Entropy distribution. The backbone dihedral angle distributions of Glycine and Proline deviate from the standard Ramachandran plot and are also fitted well by the Fourier model.

For comparison, we also generated backbone conformations from the physical force field alone and applied the same fitting procedure as for the data collected from the database. The resulting distributions are shown in Figure S3. Although the distributions have similar outlines they differ greatly in detail. Generally, the φ/ψ distributions resulting from the force field are broader and populate dihedral angles corresponding to the canonical secondary structure different from what is observed in high-resolution crystal structures. These differences are the effect of interactions such as hydrogen bonds that are not included in the physical potential function.

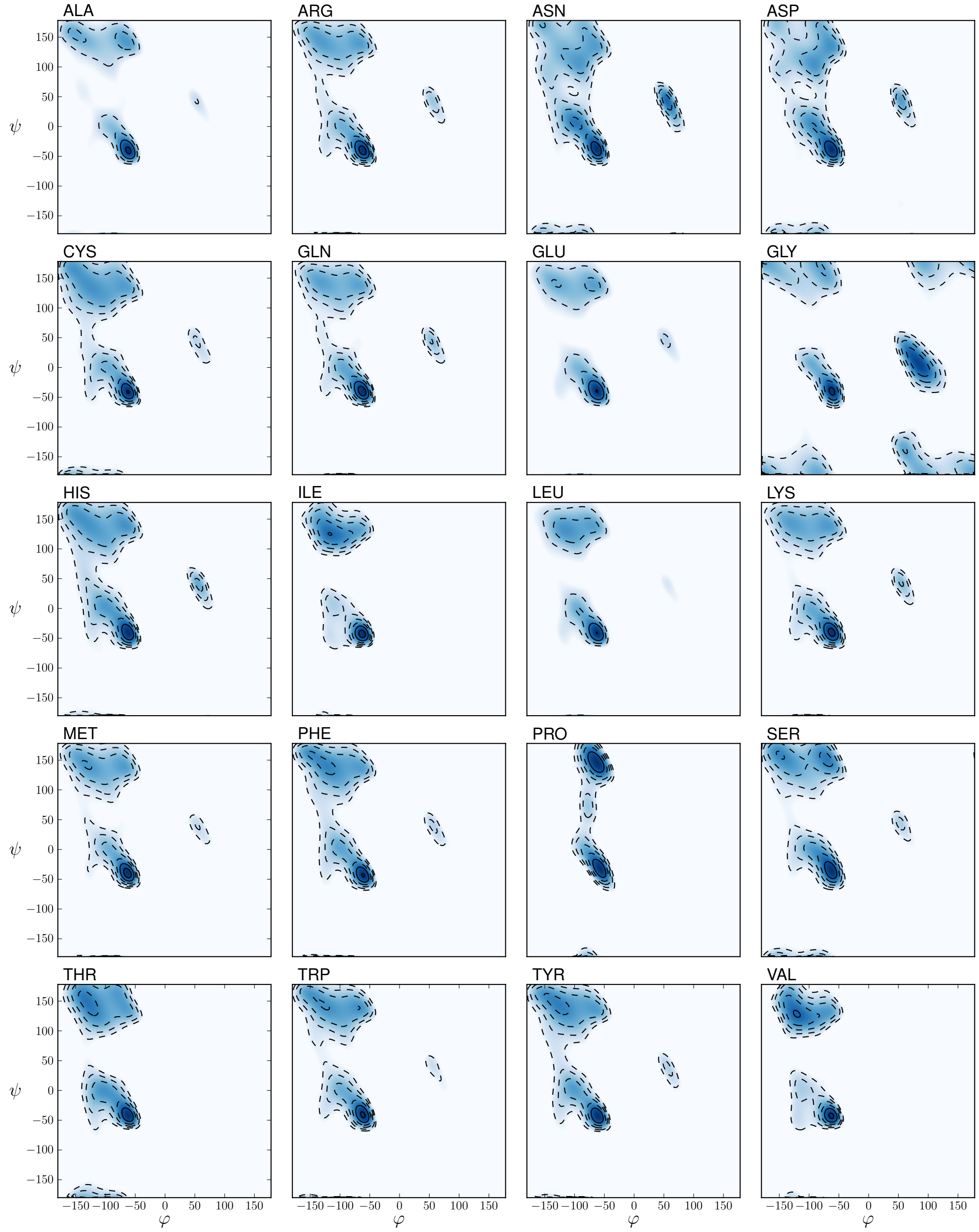

Figure S2: **Backbone dihedral angle distributions of all amino acids estimated from high-resolution crystal structures.** Heat maps of all φ/ψ distributions as approximated by the Maximum Entropy distribution outlined in this paper.

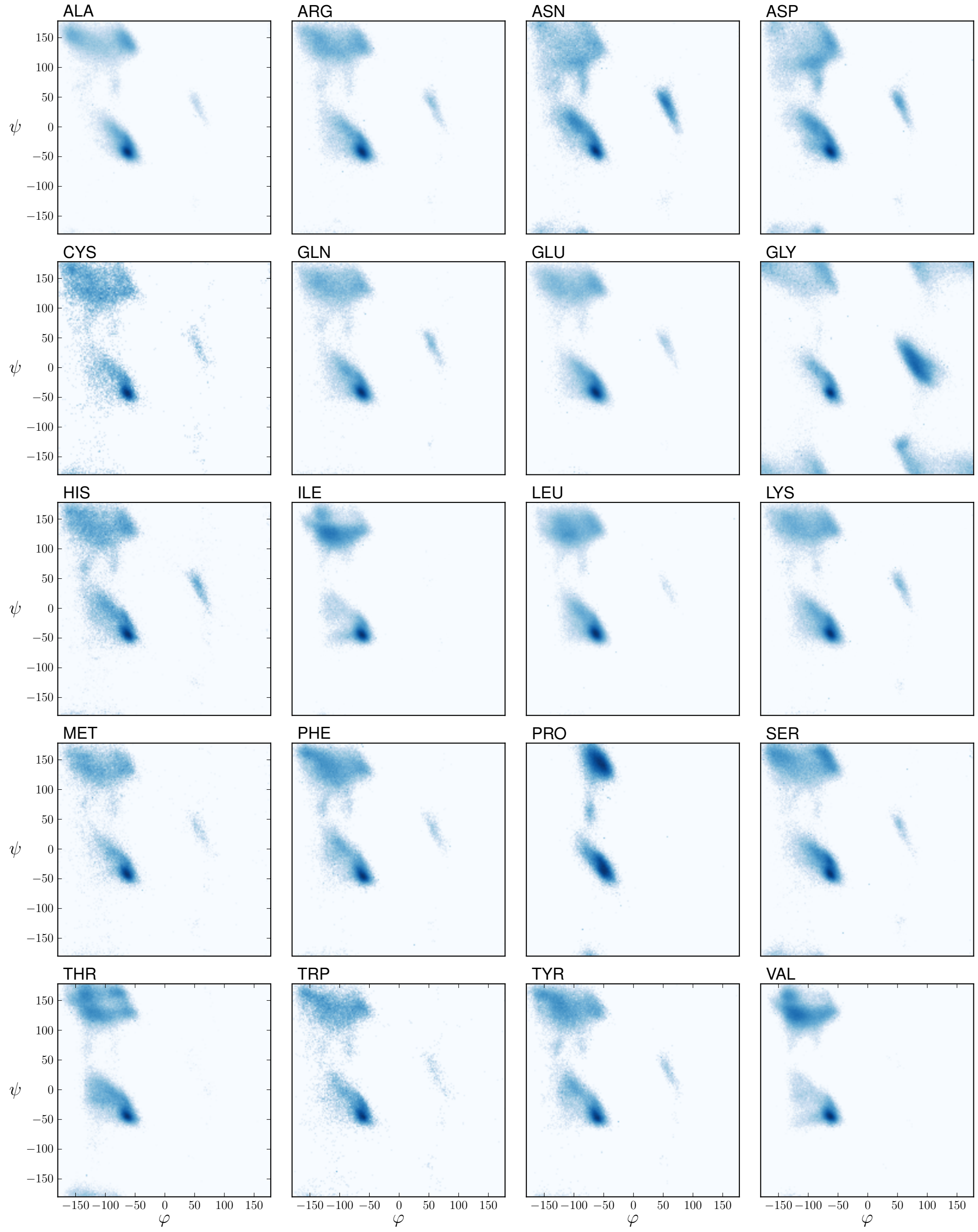

Figure S3: **Empirical Backbone dihedral angle distributions.** Histogram of the empirical φ/ψ distribution extracted from high-resolution crystal structures.

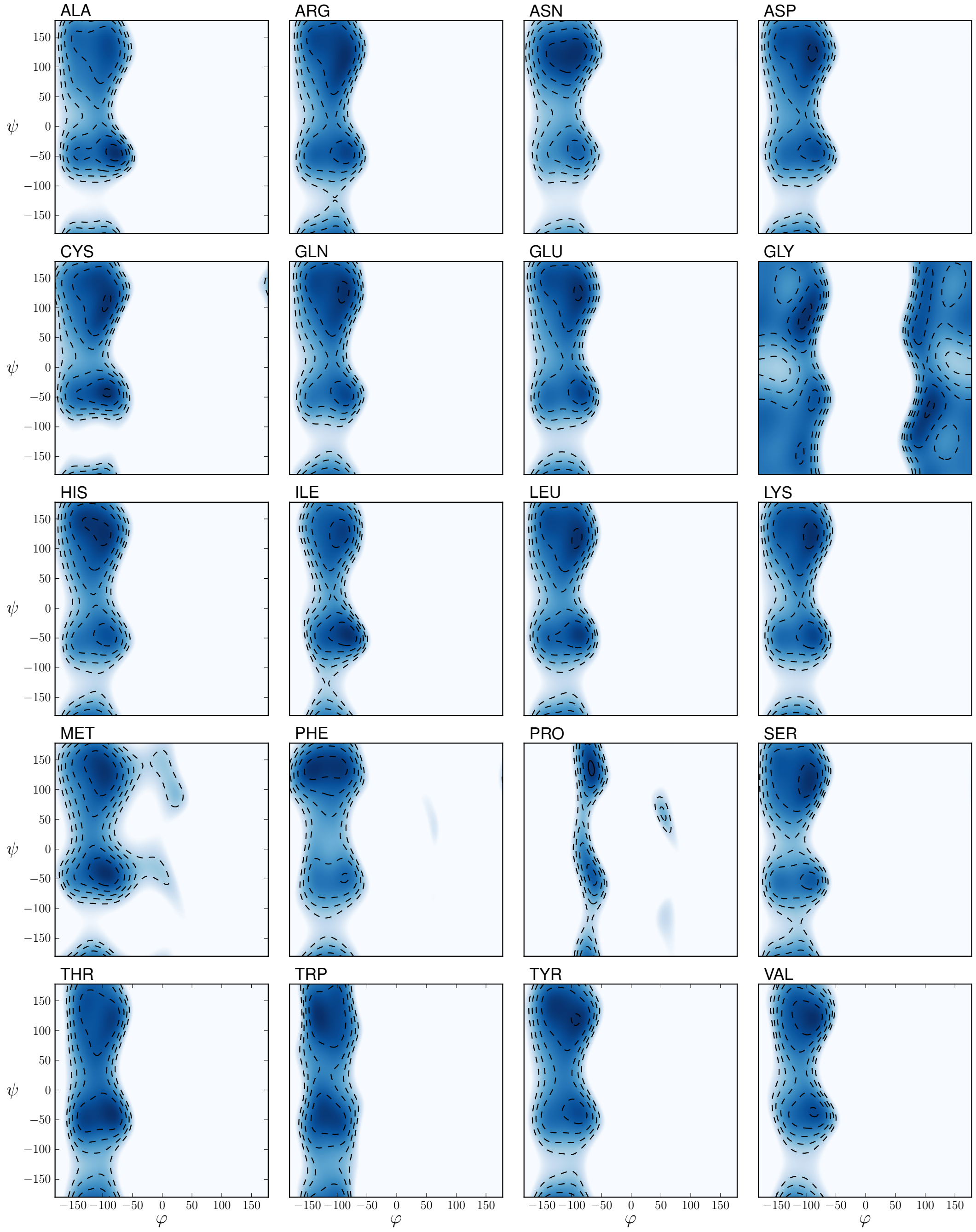

Figure S4: **Backbone dihedral angle distributions implied by the physical energy.** Heat maps of all φ/ψ distributions generated by a physical prior alone approximated by a Maximum Entropy distribution.

2 Dependence beetween quality scores, potential functions

The addition of a new potential function in structure calculation can affect all aspects of the generated structures. We assess the impact of our new backbone potential on various quality criteria in Figure S5. Figure S5 shows the mean and standard deviation of the quality scores calculated from an ensemble of 100 structures dependent on w_{rama}. None of the these scores is able to reliably select a w_{rama} that corresponds to an accurate ensemble. Taking the variation within an ensemble into account, the NQACHK score for the optimal weight structure of alpha-spectrin SH3 is well within the range of normal fluctuation and does not argue against our algorithm.

In Figure S6 we show the correlation of these quality scores for the Ubiquitin ensemble. Some of the scores seem to be highly correlated (e.g. RAMCHK and BBCCHK) and it is not clear whether it is possible to maximize all scores simultaneously. Rather we have to find a comprise between the different quality criteria, and this is exactly what our weighting scheme achieves. Furthermore, none of the scores is well correlated with the overall RMSD, supporting the notion that quality scores are not a good indicator of accuracy.

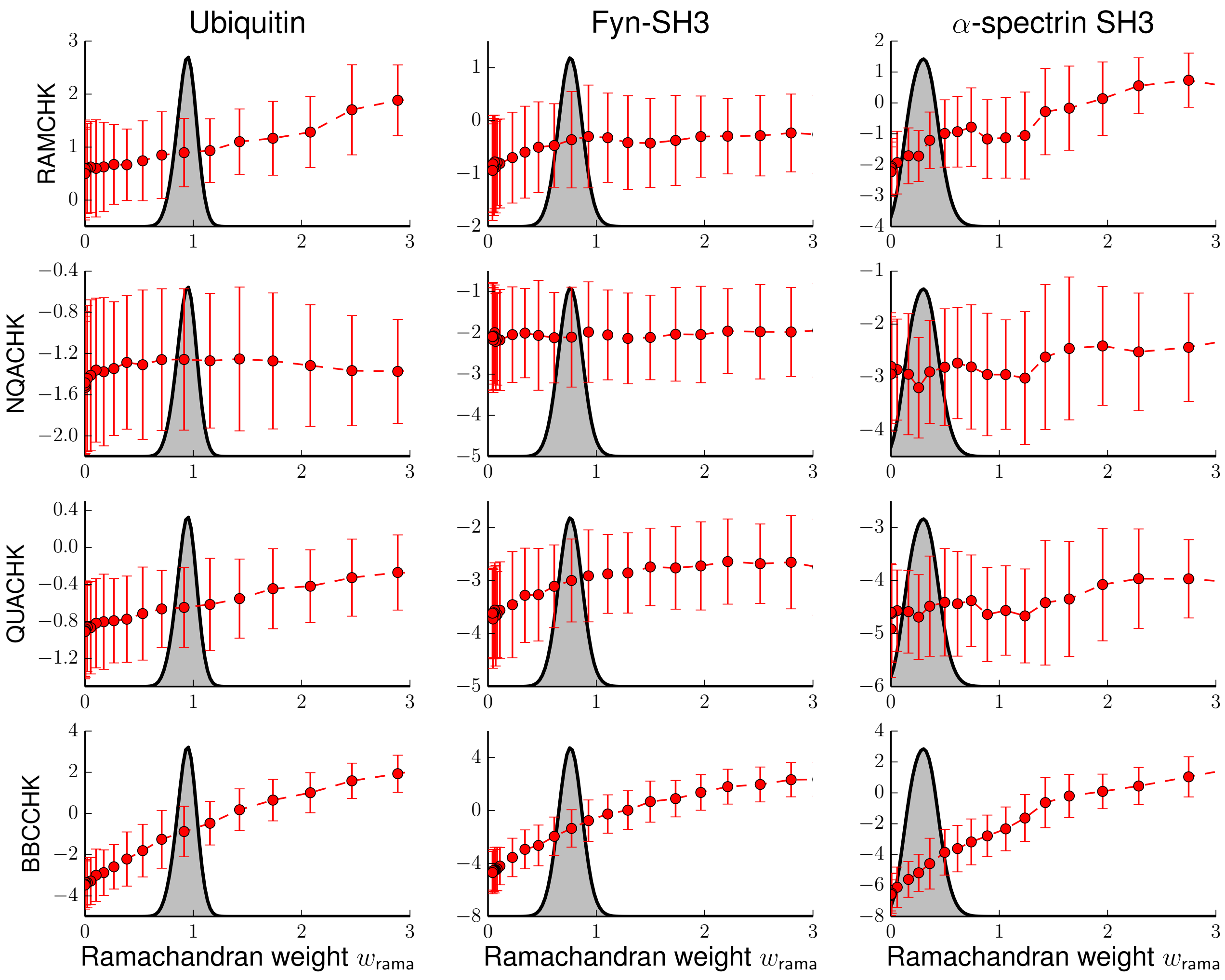

Figure S5: **Influence of the Ramachandran weight on various quality criteria.** Shown is the impact of w_{rama} on WhatCheck validation criteria. Each column reports the results for a different data set (left column: ubiquitin, middle column: Fyn-SH3 domain, right column: α-spectrin SH3 domain). Each row shows the evolution of a quality score with increasing w_{rama} (each dot marks the average over 100 structures that were randomly selected from the ISD ensemble, dashed lines are added to guide the eye). Error bars indicate one standard deviation calculated over every ensemble. The first row reports the Ramachandran appearance as assessed by RAMCHK. The second and third row show WhatCheck's packing scores. The last row reports the regularity of the backbone (BBCCK). The grey distribution indicates the model evidence $\Pr(D|w_{\text{rama}})$ as a function of w_{rama}.

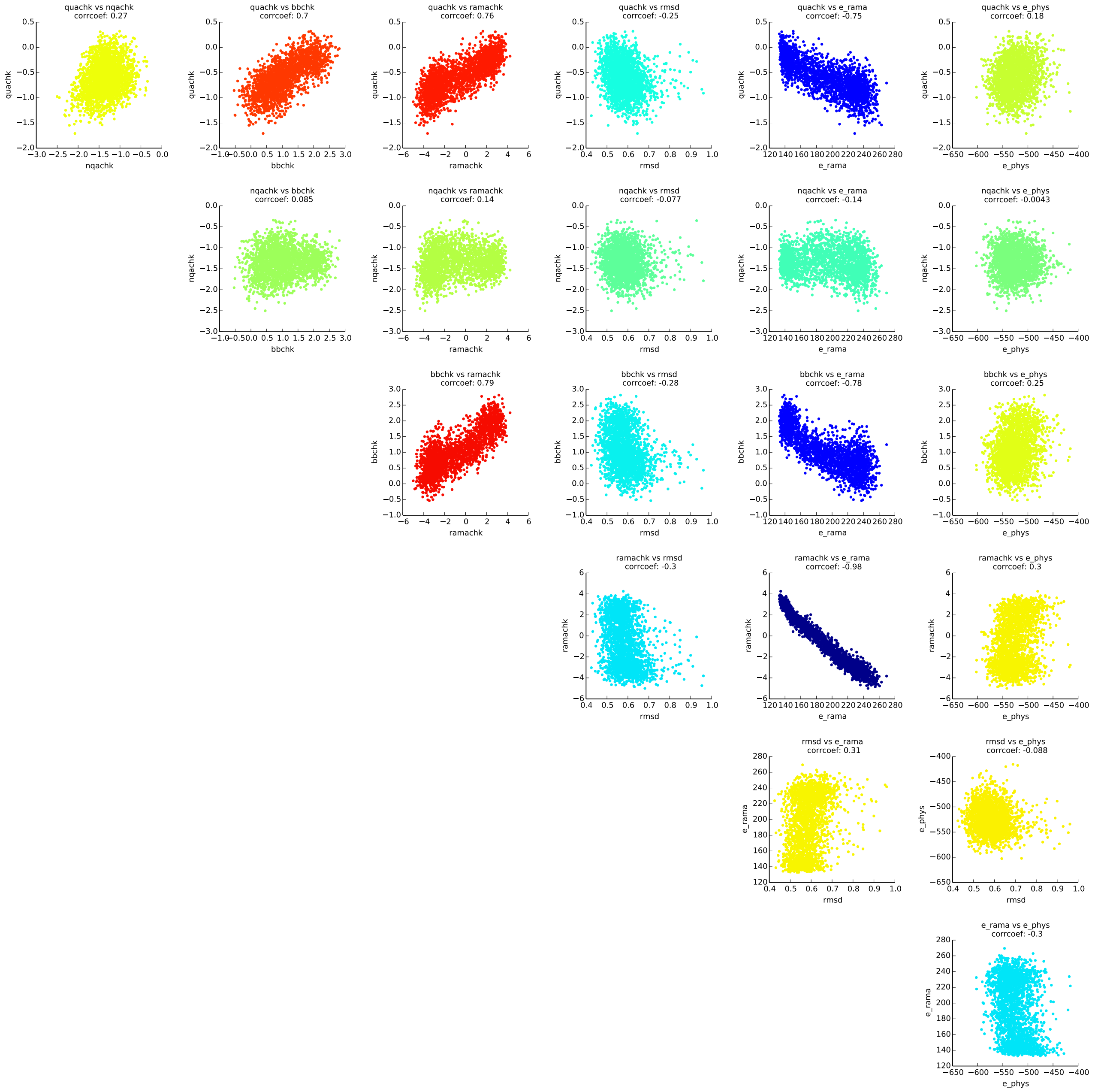

Figure S6: **Dependence between quality scores, potential functions and the RMSD.** In this figure we plot WHATCHECK qualtiy scores, our potential functions and the RMSD against each other. The color of each individual subplot represents the strength of the correlation between the two measures. The plot is based 30 ensembles comprising 100 structures each. The individual ensembles are taken from the REMC simulation of the system to calculate $\langle E_{\text{rama}} \rangle_{\text{data}}$.

3 Impact on structure ensembles from sparse and noisy NMR data

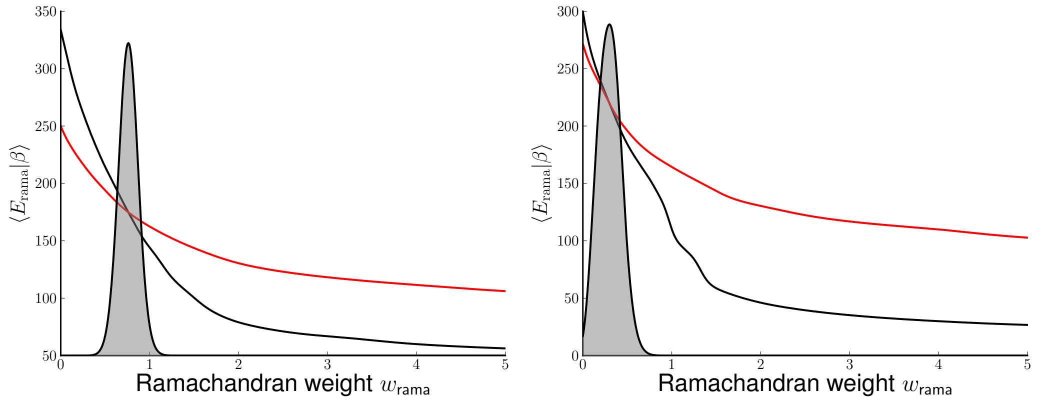

Figure S7: **Bayesian weighting with sparse and noisy NMR data.** Shown is the average backbone energy $\langle E_{\text{rama}} \rangle$ with (black) and without data (red) for the Fyn-SH3 (left) and α-spectrin SH3 domain (right). The model evidence peaks where the two curves cross.

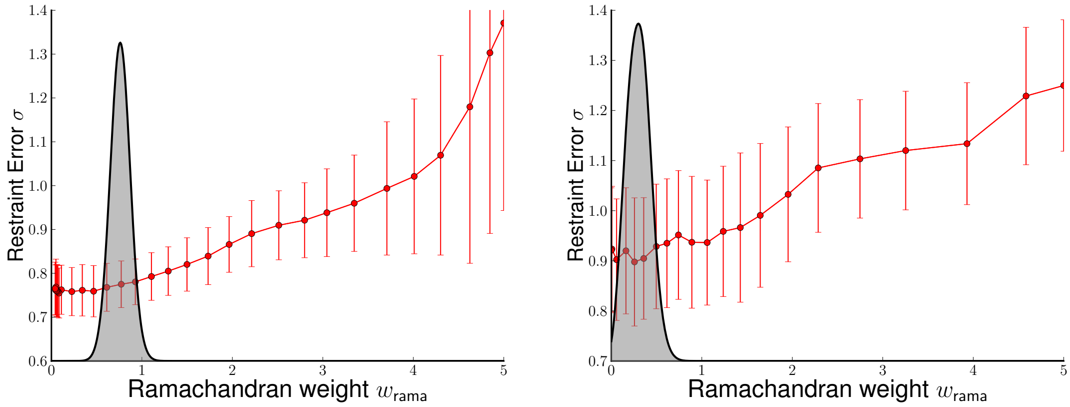

Figure S8: **Impact on the estimation of model parameters.** The red dots and error bars indicate the estimated error σ of the distance restraints for the sparse distance data (left) and the noisy restraints measured with solid-state NMR (right). The model evidence is shown as grey distribution.

Table S1: **Quality and accuracy of posterior ensembles.** The table shows various validation scores for ubiquitin, Fyn-SH3 and α-spectrin SH3. PDB entries 1ubq, 1shf and 1shg serve as reference crystal structures. The reported values are averaged over 100 conformations generated with ISD. In every sub-table, the first four rows show the Ramachandran appearance (as percentages) performed with Procheck. The next four rows list the WhatCheck Z-scores assessing the quality of packing, the dihedral angle statistics and the regularity of the backbone. The accuracy of the ensemble is measured as Cα RMSD between the crystal structure and individual ensemble members or the ensemble mean.

	**repulsive only**	**repulsive/attractive**	**repulsive/attractive and Ramachandran**	**X-ray**
**ubiquitin (1d3z)**				
Procheck				
Core	82.1 ± 3.7	85.2 ± 3.0	93.2 ± 2.0	95.5
Allowed	16.0 ± 3.6	14.3 ± 3.2	6.8 ± 2.0	4.5
Generous	1.3 ± 1.2	0.5 ± 0.8	0.0 ± 0.2	0.0
Disallowed	0.6 ± 0.7	0.0 ± 0.2	0.0 ± 0.0	0.0
WhatCheck				
RAMCHK	-3.54 ± 0.63	-3.07 ± 0.57	1.01 ± 0.46	0.849
QUACHK	-1.36 ± 0.53	-0.60 ± 0.28	-0.09 ± 0.22	1.19
NQACHK	-1.91 ± 0.32	-1.27 ± 0.31	-0.98 ± 0.30	-1.662
BBCCHK	0.84 ± 0.53	0.71 ± 0.41	1.15 ± 0.34	1.98
Accuracy				
RMSD [Å]	0.67 ± 0.08	0.60 ± 0.06	0.55 ± 0.05	–
RMSD (mean) [Å]	0.56	0.50	0.43	–
**Fyn-SH3 (1zbj)**				
Procheck				
Core	61.6 ± 6.5	59.1 ± 7.7	91.2 ± 2.9	98.0
Allowed	35.2 ± 6.8	34.4 ± 7.2	8.8 ± 2.9	2.0
Generous	3.1 ± 2.0	5.5 ± 3.5	0.0 ± 0.3	0.0
Disallowed	0.1 ± 0.4	1.0 ± 1.8	0.0 ± 0.0	0.0
WhatCheck				
RAMCHK	-5.51 ± 0.73	-5.73 ± 0.85	-1.82 ± 0.76	-0.31
QUACHK	-8.90 ± 0.61	-4.23 ± 0.82	-3.08 ± 0.39	-0.78
NQACHK	-5.81 ± 0.62	-2.50 ± 0.81	-1.86 ± 0.59	2.71
BBCCHK	-1.43 ± 0.44	-1.60 ± 0.52	-0.39 ± 0.46	-1.12
Accuracy				
RMSD [Å]	2.65 ± 0.38	1.97 ± 0.37	1.53 ± 0.20	–
RMSD (mean) [Å]	2.470	1.38	1.10	–

TableS1 – continued from previous page

	repulsive only	repulsive/attractive	repulsive/attractive and Ramachandran	X-ray
**α-spectrin SH3 (1m8m)**				
Procheck				
Core	48.2 ± 6.9	53.2 ± 6.9	93.0 ± 3.4	95.5
Allowed	43.2 ± 7.2	40.9 ± 7.2	7.0 ± 3.4	4.5
Generous	7.6 ± 2.9	5.0 ± 2.9	0.0 ± 0.2	0.0
Disallowed	1.0 ± 1.3	0.9 ± 1.3	0.0 ± 0.0	0.0
WhatCheck				
RAMCHK	-6.84 ± 0.68	-6.59 ± 0.63	-0.37 ± 0.73	-0.90
QUACHK	-7.72 ± 0.50	-4.98 ± 0.54	-4.26 ± 0.54	1.19
NQACHK	-5.00 ± 0.51	-3.10 ± 0.61	-2.68 ± 0.69	2.83
BBCCHK	-2.46 ± 0.42	-2.31 ± 0.51	0.02 ± 0.65	-0.14
Accuracy				
RMSD [Å]	3.43 ± 0.384	2.95 ± 0.33	2.72 ± 0.41	–
RMSD (mean) [Å]	2.59	2.40	2.17	–

References

H. Gong, Y. Shen, and G. D. Rose. Building native protein conformation from NMR backbone chemical shifts using Monte Carlo fragment assembly. *Protein Sci.*, 16:1515–1521, 2007.

J. Kuszewski and G. M. Clore. Sources of and solutions to problems in the refinement of protein NMR structures against torsion angle potentials of mean force. *J. Magn. Reson.*, 146:249–254, Oct 2000.

K. V. Mardia, C. C. Taylor, and G. K. Subramaniam. Protein bioinformatics and mixtures of bivariate von Mises distributions for angular data. *Biometrics*, 63:505–512, Jun 2007.

W. Boomsma, K.V. Mardia, C.C. Taylor, J. Ferkinghoff-Borg, A. Krogh, and T. Hamelryck. A generative, probabilistic model of local protein structure. *Proc. Natl. Acad. Sci. USA*, 105:8932–8937, 2008.

D. Ting, G. Wang, M. Shapovalov, R. Mitra, M. I. Jordan, and R. L. Dunbrack. Neighbor-dependent Ramachandran probability distributions of amino acids developed from a hierarchical Dirichlet process model. *PLoS Comput. Biol.*, 6(4):e1000763, Apr 2010.

E. T. Jaynes. Information Theory and Statistical Mechanics. *Phys. Rev.*, 106:620–630, 1957.

A. Pertsemlidis, J. Zelinka, J. W. Fondon, R. K. Henderson, and Z. Otwinowski. Bayesian statistical studies of the Ramachandran distribution. *Stat Appl Genet Mol Biol*, 4:Article35, 2005.

R. L. Dunbrack. Rotamer Libraries in the 21st Century. *Curr. Opin. Struct. Biol.*, 12(4):431 – 440, 2002.

W. H. Press, B. P. Flannery, S. A. Teukolsky, and W. T. Vetterling. *Numerical Recipes: The Art of Scientific Computing*. Cambridge University Press, Cambridge UK, 1989.

G. Schwarz. Estimating the dimension of a model. *The Annals of Statistics*, 6:461–464, 1978.
